# Dentists’ level of stress and used coping strategies during COVID-19

**DOI:** 10.1192/j.eurpsy.2023.1147

**Published:** 2023-07-19

**Authors:** K. S. Serota, I. Kovács, A. Balint, K. Nagy

**Affiliations:** 1 Doctoral School of Clinical Medicine, Albert Szent-Gyögyi Medical School, University of Szeged; 2 Department of Psychiatry, Albert Szent-Györgyi Medical School, University of Szeged; 3 Department of Psychiatry, Albert Szent-Gyögyi Medical School, University of Szeged; 4 Department of Oral Surgery, Faculty of Dentistry, University of Szeged, Szeged, Hungary

## Abstract

**Introduction:**

COVID-19 has increased the levels of psychological stress experienced by the dental team, and higher level of constant stress negatively impacts mental health.

**Objectives:**

The study aimed to 1) assess dentists’ level of stress and compare it to normal population data; 2) identify the hierarchy of coping strategies chosen by dentists and their perception of those chosen by team members to manage psychological stress caused by the pandemic; and 3) to ascertain the effects of these coping strategies on dentists’ higher stress level.

**Methods:**

Data from an electronic test battery comprising of general demographic and dental-related variables was collected from 182 licenced Hungarian dentists at the outset of the pandemic. Responses to an empirical series of questions regarding their perceived level of stress, choice of interventional coping skills and their perception of those used by team members were recorded.

**Results:**

Dentists’ level of stress was significantly lower than the stress level measured in a Hungarian normal population (*t* (386)=-2.227, *p*=0.027), while financial status has a moderating effect (*F*(3,176)=4.851, *p*=0.003). The hierarchy of coping strategies chosen by the dentist indicated that physical activity and exercise, particularly in groups settings (M=4.78, SD=0.463), and socialization with family (M=4.72, SD=0.626) were the most effective coping management strategies, superior to financial compensation, shifting work patterns, systems level change, and decisions within the team structure. Inclusionary strategies with family (M=4.64, SD=0.587), participating in individual leisure activities (M=4.49, SD=0.621) and socializing with friends (M=4.44, SD=0.825) were seen by dentists as more important to team members. Regression analysis was used to ascertain whether the use of these coping strategies increased the likelihood of having higher levels of perceived stress. The model was significant (*F*(4,169)=8.292, *p*≤0.001) with R^2^ of 16.4%. Older age (B=-0.179, S.E.=0.050, *t* =-3.582, *p*≤0.001), gender (B=4.214, S.E.=1.423, *t*=2.961, *p*=0.004), active participation in developing COVID-19 protocols (B=-1.619, S.E.=0.575, *t* =-2.815, *p*=0.005) and socialization with family (B=-2.108, S.E.=1.058, *t* =-1.993, *p*=0.048) were the most effective coping mechanisms for having lower levels of perceived stress.

**Conclusions:**

Our study provided insights into the value of importance attributed to perceived stress and a series of coping strategies used by the respondents and their perception of value ascribed to the same series by their team members. Active participation both in family life and in professional environment proved to be protective in such a highly stressful time like the COVID-19 pandemic.

**Disclosure of Interest:**

None Declared

